# Balancing patients’ benefits and risks in computed tomography: a European Society of Radiology ‘EuroSafe Imaging’ viewpoint

**DOI:** 10.1007/s00330-025-11911-8

**Published:** 2025-08-09

**Authors:** John Damilakis, Claudio Granata, Elmar Kotter, Boris Brkljacic, European Society of Radiology (ESR)

**Affiliations:** 1 Department of Medical Physics, School of Medicine, University of Crete, Crete, Greece; 2 Department of Paediatric Radiology, Institute for Maternal and Child Health, IRCCS “Burlo Garofolo”, Trieste, Italy; 3 Department of Diagnostic and Interventional Radiology, Medical Center University of Freiburg, Faculty of Medicine, University of Freiburg, Freiburg, Germany; 4 University Hospital Dubrava, University of Zagreb School of Medicine, Zagreb, Croatia; 5 European Society of Radiology (ESR), Vienna, Austria

**Keywords:** CT, Cancer, Eurosafe

## Abstract

**Abstract:**

Every physician seeks to do more good than harm. A surgeon operates with the expectation that the benefits of the operation outweigh the risks associated with the surgery, leaving the patient in a better condition than before the procedure. Radiologists provide enormous benefits for patients—identifying diseases that could not otherwise be diagnosed or staging disease severity to allow for the most appropriate treatments to be undertaken. However, like any intervention, radiological studies are associated with risks; ionizing radiation may not be as apparent as a surgical site infection to patients or even other healthcare providers, but it carries inherent and cumulative risk and portends real hazard for patients. This article explores the responsibility of radiologists to champion appropriate imaging selection through rigorous justification of studies and protocol optimization when imaging is indicated.

**Key Points:**

- *Irradiating patients for CT imaging provides tremendous diagnostic value, but is associated with an increased risk of cancer.*
- *The risk of CT-associated malignancies should be discussed with patients in an understandable way, considering individual- and population-level hazard.*
- *Strict application of the ALARA principle allows for maximizing benefits from imaging studies while minimizing harm.*

## CT-associated cancer burden

Computed tomography (CT) has become an indispensable diagnostic tool. Alongside its clear clinical benefits, CT imaging exposes patients to ionizing radiation. A new study has found that about 103,000 future cancers are projected to result from the CT scans performed in the U.S. in 2023 [1]. This corresponds to roughly 5% of all new cancer diagnoses annually if current practices persist. These findings update and far exceed a 2009 estimate, which projected ~29,000 cancers from CT scans in 2007, mainly reflecting the growth of CT utilization [2].

Large-scale cohort studies have observed an increased cancer incidence following CT exposure. For instance, retrospective studies have shown that children who underwent CT scans had higher rates of leukemia and brain tumors later in life [3]. These epidemiological findings, while statistically significant, faced questions about bias (e.g., children who needed CT scans might have other risk factors) and the challenge of detecting a small signal (radiation-induced cancers) against the higher “noise” of background cancer incidence. Data suggest that for every 10,000 children who undergo CT scanning today, about 1–2 might develop a radiation-attributable leukemia or lymphoma within the next 12 years [3]. While the absolute risk per child is very small, this empirical confirmation of risk at typical CT dose levels is important. These real-world findings bolster the credibility of model-based projections, and they extend earlier research by demonstrating risk with greater statistical power and methodological care.

It is also important to recognize that evidence for CT-related cancer risk in adults has been less direct. Adults undergo far more CT scans than children, but showing a definitive excess cancer risk epidemiologically is difficult because their baseline cancer risk is higher, and the latency for solid tumors can be decades.

## Implications for clinical practice and radiation safety

Findings related to cancer risk carry important implications for how the medical community approaches the use of CT. First and foremost is the reaffirmation of the core principles of radiation protection: justification and optimization. Every CT scan may not be risk-free—even if the risk to any individual patient is very low—and thus every scan should have a clear clinical indication. A recent study about the justification of CT examinations, conducted in seven EU member states, has shown large variability in the appropriateness of CT exams and the necessity to strictly adhere to principles of justification [4]. Initiatives such as the European Society of Radiology (ESR) EuroSafe Imaging Campaign, Image Gently and Image Wisely have sought to educate healthcare personnel about appropriate use of imaging and dose reduction techniques. It is notable that in pediatric practice, heightened awareness has already led to declines or substitution of CT in many settings (e.g., greater use of ultrasound or MRI in cases of appendicitis and trauma in children). In adult medicine, however, CT utilization remains high and, in some areas, continues to grow. Radiologists and referring physicians must work together to ensure that CT scans are ordered only when the expected clinical benefit outweighs the radiation risk.

Equally critical is optimizing how scans are performed when they are justified. Modern CT technology offers many tools—automated exposure control, iterative image reconstruction, tailored protocols—that can substantially lower the dose per scan without sacrificing diagnostic accuracy. Radiology departments should continually update protocols in line with the latest dose-saving techniques. Strict application of the ALARA principle (As Low As Reasonably Achievable) is essential. In practice, this might mean double-checking if an alternative exam without radiation could answer the clinical question, or using optimized protocols that, for example, limit the scanned region and phases to the minimum necessary. It also means fostering a culture where radiologists feel empowered to question or suggest modifications to scan requests that seem unjustified or excessively frequent.

## Communicating risk without causing alarm

One challenging question is how to communicate the risks to patients and the public in a balanced manner. On one hand, transparency about medical radiation exposure is important; patients have a right to know that a CT scan, while providing important diagnostic information, also carries a very small potential to contribute to cancer risk in the future. On the other hand, these risks must be put into proper perspective to avoid undue fear. The absolute risk to any single patient from one CT scan is very small. By contrast, the immediate medical risk of not scanning a child with serious symptoms (and thereby missing a critical diagnosis) could be far higher. It is crucial that patients understand that CT scans are generally safe, and very often lifesaving, when used appropriately. Misinterpretation of studies on radiogenic risk associated with CT and other X-ray examinations in the media could inadvertently lead some people to equate a CT scan with a near-certain cancer outcome, which is absolutely not the case [5]. Health providers must therefore walk a fine line, aided by frameworks like the WHO’s evidence-based communication tools for discussing imaging risks [6], raising awareness of cumulative population risk without deterring individuals from needed imaging.

Patients can be told that the added potential risk from a single CT is very low, but since millions of scans are done, we as a society want to eliminate any exposures that are not necessary. Another strategy is to emphasize what is being done to ensure safety; patients may find it reassuring to hear that the hospital actively tailors doses, uses state-of-the-art low-dose imaging systems, and follows guidelines to avoid unwarranted scans. Such communication keeps confidence in medical imaging high while also respecting the patient’s right to be informed. In essence, the message should be: “We will only do this CT if it’s needed, and if we do it, we will use the lowest possible dose because we are aware of even the very small risks involved.” This kind of nuanced dialogue can prevent misinterpretation and maintain trust.

## Balancing benefits and risks: the path forward

As X-ray imaging technology advances and spreads, the responsibility to manage its downsides grows commensurately. Recent findings about risks largely reinforce the existing paradigm of ALARA and careful benefit-risk analysis for each study. They also challenge any complacency in the medical community. If there are still pockets of routine CT overuse, non-adherence to imaging referral guidelines, or outdated scanning protocols delivering higher radiation than necessary, now is the time for those to be addressed.

Moving forward, further research should continue to refine our risk estimates and guide safety improvements. On the technical front, development of newer imaging techniques (e.g., low-dose photon-counting CT or MRI/ultrasound alternatives), AI-powered radiation protection optimization, and AI-assisted image reconstruction holds promise to maintain diagnostic efficacy with even less radiation [7]. Importantly, enhanced tracking of individual patients’ cumulative dose across healthcare systems could enable more personalized assessments and inform decision-making [8].

In conclusion, the projected lifetime cancer risks from current CT use highlight a classic public health precaution: widespread exposure to a small individual risk can produce a considerable population-level impact. A balanced interpretation of relevant studies is essential. It should neither incite an undue alarm nor be ignored. Instead, it should galvanize the medical community to double down on best practices: ordering CT scans thoughtfully, relentlessly optimizing scans, and communicating openly yet carefully about the risks. By doing so, the principle that underpins all of medicine—*primum non nocere*, “first, do no harm”—is honored while still harnessing the lifesaving benefits of modern imaging technology.

## Abbreviations

ALARA: As Low As Reasonably Achievable
ESR: European Society of Radiology
